# Ovarian expression of functional MTTP and apoB for VLDL assembly and secretion in chickens

**DOI:** 10.1016/j.psj.2025.104993

**Published:** 2025-03-06

**Authors:** Yu-Hui Chen, Petnamnueng Dettipponpong, Mei-Ying Sin, Ling-Chu Chang, Chuen-Yu Cheng, San-Yuan Huang, Rosemary L. Walzem, Hsu-Chen Cheng, Shuen-Ei Chen

**Affiliations:** a Department of Animal Science, National Chung Hsing University, Taichung 40227, Taiwan; b Department of Life Sciences, National Chung Hsing University, Taichung 40227, Taiwan; c Center for Molecular Medicine, China Medical University Hospital, Taichung 404327, Taiwan; d Research Center for Cancer Biology, China Medical University, Taichung 40402, Taiwan; e Cancer Biology and Precision Therapeutics Center, China Medical University, Taichung 40402, Taiwan; f Department of Animal Science and Biotechnology, Tunghai University, Taichung 407224, Taiwan; g The iEGG and Animal Biotechnology Center and Rong Hsing Research Center for Translational Medicine, National Chung Hsing University, Taichung 40227, Taiwan; h Department of Poultry Science, Texas A&M University, College Station, TX 77843, USA; i i-Center for Advanced Science and Technology (iCAST), National Chung Hsing University, Taichung 40227, Taiwan

**Keywords:** Chickens, Ovary, VLDL secretion, Microsomal triglyceride transfer protein, Apolipoprotein B

## Abstract

In mammals, tissues other than liver and intestine are known to possess functional MTTP (microsomal triglyceride transfer protein) and apoB (apolipoprotein B) capable of VLDL (very low-density lipoprotein) assembly. Birds are oviparous and possess unique capabilities in lipid biology to accommodate yolk formation through massive deposition of hepatically assembled yolk-targeted VLDLy into ovarian follicles. Following identifications of MTTP and ApoB expression within chicken ovarian stroma, granulosa, theca, and epithelial cells of various classes of follicles, we sought to define the functionality of ovarian MTTP and ApoB in VLDL assembly. In situ hybridization analysis found that ApoB transcripts are most abundant in thecal layers, whereas immunohistochemistry showed that MTTP predominates in the granulosa layers. MTTP lipid transfer activity was greater in small yellow follicles than in hierarchical follicles. Metabolic labeling, electron microscopy, and Western blot studies confirmed the functionality of ovarian apoB and MTTP as newly assembled VLDL around 50-200 nm in diameter and lacking ApoVLDL-II dissimilar to VLDLy, were secreted from cultured follicular cells. Lomitapide and the ApoB-antisense oligonucleotide Mipomersen dose-dependently decreased MTTP activity and VLDL-apoB secretion from cultured follicular cells, while oleate addition or acute heat stress enhanced VLDL-apoB secretion. Ultrastructural images showed VLDL assembly and trafficking toward the secretion route. The findings support the notion that VLDL assembly and secretion within avian ovarian tissues functions as a protective mechanism against fuel and physical stressors to secure follicle development and/or nutritional quality control of yolk for embryo development.

## Introduction

The liver and intestine are the primary tissues known to possess functional MTTP (microsomal triglyceride transfer protein) and synthesize Apolipoprotein B (ApoB) to assemble VLDL (very low-density lipoprotein)(Wetterau and Zilversmit, 1984; Raabe et al., 1999; Hussain et al., 2003). A key alteration in VLDL-biology in laying hens is the incorporation of Apolipoprotein VLDL-II (ApoVLDL-II) into hepatically assembled VLDL reducing particle diameter to ∼30 nm and conferring lipase resistance; both changes facilitate VLDL passage across follicle layers to reach the oolemma for yolk deposition (Perry et al., 1984) and preserve its triacylglycerol (TAG) for embryonic use (Schneider et al., 1990; Boyle-Roden and Walzem, 2005). This unique VLDL is designated as VLDLy to indicate its yolk targeting for TAG delivery (Walzem et al., 1999). Prior to identification of the receptor-mediated uptake of VLDLy by LR8- receptor (Barber et al., 1991), Bensadoun's group reported high rates of LPL (lipoprotein lipase, EC no. 3.1.1.34) activity in ovarian tissues and hypothesized that lipid deposition in yolks may occur through a process similar to that found in adipose tissue (Benson et al., 1975).

ApoB-containing lipoproteins (apoB-LP) can serve protective as well as energy and nutrient delivery roles. For example, in addition to liver and intestine, the failing human heart was found to possess MTTP activity and synthesize ApoB capable for VLDL assembly and secretion; perhaps as a safety mechanism to prevent accumulation of excess fatty acids in the tissue (Borén et al., 1998). Other works further supported the possibility that VLDL assembly and secretion in tissues/organs such as heart and kidney act to maintain lipid homeostasis, protect the tissue/organ against metabolic derangements due to TAG accumulation, oxidative stress, lipotoxicity, and even functional compromise (Nielsen et al., 1998; Bartels et al., 2009; Yokoyama et al., 2004; Krzystanek et al., 2010). Chickens also assemble VLDL in the kidney, which may reflect a reabsorption mechanism to capture lipid substrates present in the glomerular filtrate (Walzem et al., 1999).

VLDL assembly also occurs in the placenta and yolk sac visceral endoderm of mammals suggesting a general role for the lipid delivery to the fetus and embryo, and moreover a mechanism of the early embryo to prevent excessive accumulation of lipids deleterious to the developing embryo (Farese et al., 1996; Terasawa et al., 1999; Madsen et al., 2004). The liver and small intestine also predominate in apoB synthesis and MTTP activity responsible for apoB-LP assembly in birds, with the distinction that birds only make ApoB100 (apolipoprotein B-100) (Griffin et al., 1982; Kirchgessner et al., 1987; Tarugi et al., 1990; Ivessa et al., 2013). Chicken yolk sac membrane (YSM) also synthesizes ApoB, and reportedly contributes to plasma VLDL in the developing embryo (Kanai et al., 1996), albeit the contribution of preexisting VLDL in yolk lessens the conclusion since YSM epithelial cells also express considerable VLDL/LDL receptors (Hermann et al., 2000). The YSM epithelial cells in developing eggs were also shown to express functional MTTP and associated lipid transfer activity (Ivessa et al., 2013; Eresheim et al., 2014). However, these earlier studies did not demonstrate functional VLDL assembly processes. Leclercq et al. reported that VLDL of hens provided with free access to feed carried nearly half of NEFA (non-esterified fatty acids) found in plasma, while 26 h after feed removal the NEFA concentration did not decrease but shifted its association from VLDL to albumin (Leclercq et al., 1979). Such findings suggest that lipid substrates deposited into the yolk can vary and may require mechanisms to limit free fatty acid concentrations in yolk.

We were the first to use microarray and proteomic approaches to show that the small yellow follicles (SYFs) in the ovary of hens responded to acute heat stress by upregulating transcript expression and protein synthesis of genes needed for lipid cargo export (Cheng et al., 2018a; 2018b). These findings suggest that VLDL assembly might occur within the avian ovary, which might serve as a protective mechanism to secure follicle development and a quality control of yolk lipids for successful embryo development. The present study sought to determine whether the presence of ApoB and MTTP functions to assemble VLDL within avian ovarian tissues and whether the ovarian secretion of VLDL responds to physiological alterations.

## Materials and methods

### Animals

The Institutional Animal Care and Use Committee (IACUC) of National Chung Hsing University, Taiwan, reviewed and approved all chicken husbandry and experimental procedures (IACUC Permit NO. 109-134). Leghorn hens aged 35 to 55 weeks old of a strain that reliably lays eggs until 100 weeks of age were maintained for sampling throughout the study. Follicle preparations from differently aged hens performed similarly. To ensure that hens used in the studies possessed ovaries of similar functions, only those hens that had laid an egg each day for a minimum of 3 days were used for sampling. Hepatocytes from 1-day-old chicks of the same strain were used as a comparison cell type for MTTP activity and ultrastructure analysis of secreted VLDL.

### Follicle tissue collection and isolation of ovarian follicle cells and hepatocytes

Various classes of follicles including the hierarchical follicles (F1 to F5, 1-2.5 cm in diameter), small yellow follicles (SYFs, 5-7 mm) and large white follicles (LWFs, 1-3 mm) were carefully separated from ovaries collected from hens. For whole follicle tissue collection, the yolk of hierarchical follicles and fluid within SYFs, and LWFs were first removed by syringe aspiration. Follicles were then cut off and gently rinsed with PBS to flush out the residual yolk or fluid. The remaining intact follicle walls and ovarian stroma (OS) were then dissected, minced into pieces (approximately 3 mm2). For culture studies with whole follicle cells, the pieces from F2 follicles, SYFs, and LWFs were further dispersed by type-2 collagenase (200 U/mL in M199-HEPES medium) at 37 °C for 40 min. The incompletely digested tissue lysates were pelleted by centrifugation at 50 × g for 5 mins prior to removal of supernatants containing dispersed cells. Cells were pelleted by centrifugation and the pellets were re-suspended in M199 medium containing 10 % FBS for VLDL secretion and gene expression studies. F1 follicles were not used in these studies because oviposition time was not precisely recorded and variations due to, for example, F1 progesterone secretion, could not be excluded.

Hierarchical F2 to F5 cells were used for culture studies with specific cell type. Granulosa cell (GC) layers were isolated according to the method of Gilbert et al. (1977). Briefly, the GC layer was isolated from the theca (TH) and superficial epithelial (EP) layers of the follicle wall by carefully peeling off the individual tissue layers using sharp forceps. Collected GC, TH, and EP layers were rinsed twice with PBS and dispersed in M199-HEPES medium containing 200 U/mL type-2 collagenase and 0.3 mg/mL trypsin inhibitor (Sigma-Aldrich, St Louis, MO, USA) at 37 °C for 20 min. Dispersed cells of individual type were suspended in M199 medium (Sigma-Aldrich) containing 10 % FBS (fetal bovine serum, Thermo Fisher Scientific Waltham, MA, USA) for cell culture, gene expression, and MTTP activity studies.

Chick hepatocytes were isolated using a two-step collagenase perfusion method (Lee et al., 2013a). Isolated hepatocytes were used for MTTP activity analysis immediately, or cultured as described in individual experiments for VLDL secretion analysis.

### Cell cultures

Gelatin-coated (150 μg gelatin/cm^2^) 6-well plates (Sigma-Aldrich) were seeded with isolated follicle cells or chick hepatocytes (1 × 10^6^ cells/well) in medium (containing 10 mM HEPES, 18 mM NaHCO_3_, 100 U/mL penicillin, 100 pg/mL streptomycin sulfate, and 10 % fetal bovine serum, pH 7.4) at 37 °C under 5 % CO_2_/95 % air for 2 days. M199 and DMEM medium were used for culture studies with follicle cells or chick hepatocytes, respectively. Cells were then replenished with new medium and grown to 85 % confluence for VLDL and ApoB secretion studies. Newly added medium contained additional GSH (glutathione, 50 μg/mL), BHT (butylated hydroxytoluene, 5 μg/mL), PMSF (phenylmethylsulfonylfluoride, 35 mg/mL), and protease cocktail (250 μg/mL, Roche, Basel, Switzerland) prior to overnight culture at 37 °C . Culture medium and cells collected the next day for VLDL isolation and/or ApoB content determination by Western blot analysis. For heat stress (HS) studies, follicle cells were grown to 85 % confluence at 37 °C prior to being subjected to 3 hrs of incubation at 42 °C prior to being returned to 37 °C for overnight culture. Control follicle cell cultures were cultured in parallel at a constant 37 °C.

Oleic acid (OA, C18:1, n-9, Sigma-Aldrich) was supplemented as a complex with 5 % fatty acid-free bovine serum albumin (BSA) (8:1, M:M; Research Organics, Cleveland, OH, USA) to a final concentration of 0.1 mM which is within a physiological range (Chen et al., 2006; Xie et al., 2012). The MTTP inhibitor Lomitapide (Sigma-Aldrich) and the apoB synthesis inhibitor Mipomersen (MedChemExpress, Monmouth Junction, NJ, USA) were supplemented in DMSO at various concentrations (from 0.1 to 10 μM and 0.5-5 μM, respectively).

### VLDL isolation

VLDL was isolated from hen plasma and culture medium as a 1.006 g/ml density fraction following centrifugation at 148,600 × g for 18 h at 14 °C (Walzem et al., 1994). VLDL fractions isolated from culture medium were further concentrated by ultrafiltration using 5 kDa MW cut-off spin-columns (Millipore, Burlington, MA, USA).

### Antibody production

Purification of chicken plasma ApoB and Apolipoprotein VLDL-II (ApoVLDL-II) was conducted according to the method from Nimpf et al. (1988). Briefly, VLDL was isolated from 1.5 mL of laying hen plasma prior to delipidation by consecutive extractions with chloroform/methanol. The residues were dried by a stream of nitrogen, resuspended in a buffer containing 10 mM Tris-HC1, 60 mM octylglucoside, pH 7.4, and incubated overnight at 4 °C with gentle agitation. Undissolved fractions containing ApoB were pelleted at 5000 × g, 4 °C, while ApoVLDL-II remained in the supernatant fraction. Pellets and supernatants were collected separately, dialyzed against distilled water, and then lyophilized. Purified ApoB and ApoVLDL-II were examined by SDS-PAGE for purity (Fig. S1) and used as an antigen to immunize mouse and rabbits, respectively. A synthetic peptide (CRKVFSTASDSSGSWF) (Thermo Fisher Scientific, Waltham, MA, USA) corresponding to the carboxy-terminal of the M subunit (97 kDa) of chicken MTTP was used as an epitope to immunize rabbits.

The synthetic MTTP-M epitope, purified ApoVLDL-II or ApoB was coupled with KLH (keyhole limpet hemocyanin) (Merck, Darmstadt, German) as a carrier protein and mixed with Freund's complete adjuvant (Sigma-Aldrich). Adult female New Zealand rabbits were used to elicit antibody production against chicken MTTP-M and ApoVLDL-II while antibody production against chicken ApoB used BALB/c mice. Antisera was purified by affinity chromatography through a protein A-sepharose CL-4B (Thermo Fisher Scientific) column to obtain IgG fraction. Immunohistochemistry and Western blot with laying hen tissues were used to validate the MTTP-M antibody specificity (Fig. S2 panel A and B).

### Immunohistochemistry

Antigen retrieval was performed as described using formalin-fixed and paraffin-embedded ovarian follicle (rinsed to be devoid of yolk) and stroma tissue sections (Liu et al., 2014). Sections were washed with PBS-Tween 20 (0.1 %) buffer 3 times, 3 min/time, blocked in 10 % bovine serum albumin (BSA) in PBS-Tween 20 buffer for 30 min, and probed with the primary rabbit antibody against chicken MTTP-M prepared as described above. The capture antibody was a peroxidase-conjugated goat antibody against rabbit IgG (Cell Signaling Technology, Danvers, MA, USA) with DAB (3,3-Diaminobenzidine) used for chromogenesis and visualization by an optical microscope (DMIRB; Leica, Wetzlar Germany). The MTTP-M antibody specificity was determined using liver and pancreas sections using an Alexa FluorTM 488 conjugated goat antibody (Cell Signaling Technology) and fluorescence microscope (DMIRB; Leica)(Fig. S2 panel A).

### In situ hybridization analysis

RNAscope® 2.5 HD Detection Reagent–BROWN kit (Advanced Cell Diagnostics, Newark, CA, USA) was used for in situ hybridization of ApoB transcripts in ovarian tissues. The RNAscope probe targeting chicken ApoB gene (92-1159 of clone NM_001044633.1 in Genbank) was custom designed (Advanced Cell Diagnostics). Fixation, embedding, target retrieval, and probe hybridization followed the manufacturer's instructions.

### Electron micrograph

The imidazole-buffered osmium tetroxide method was used to visualize VLDL assembly processes in electron micrographs (Angermüller and Fahimi, 1982). Collected follicles were pre-fixed in a modified 0.15 M cacodylate buffer containing 2.5 % glutaraldehyde, 2 % paraformaldehyde, 2 mM CaCl2, pH 7.4 for 1 hr (Hamilton et al., 1998). Follicles were then cut off, gently rinsed with fixative buffer to flush out the residual yolk, dissected into pieces, and returned to fixative buffer at 4 °C and held overnight prior to 5 × 3 min/time rinse with fixative buffer and transfer into 0.15 M cacodylate buffer containing 2 % osmium tetroxide,1.5 % potassium ferrocyanide, 2 mM CaCl2 at 4 °C for 1 hr. Thereafter, samples were transferred into 1 % thiocarbohydrazide buffer at room temperature for 20 min, 2 % osmium tetroxide buffer at room temperature for 30 min, and finally into 1 % uranyl acetate buffer at 4 °C for overnight, during which samples were rinsed 5 × 3 min/time with DDW following each round of buffer changes. Samples were stained in Walton's lead aspartate buffer (0.66 % lead nitrate, 0.4 % l-aspartic acid) at 60 °C for 30 min, rinsed 5 × 3 min/time. with DDW then dehydrated in a sequence of 20 %, 50 %, 70 %, 90 %, 100 % of 4 °C ethanol 2 times, 7 min/time, transferred into cold acetone, 4 °C, for 10 min followed by room-temperature acetone for 10 min. Treated samples were infiltrated with Spurr's resin (EMS, Hatfield, PA, USA) overnight at room temperature. The next day, samples were infiltrated in new Spurr's resin for an extra 2-hr prior to resin polymerization and sample embedment by incubation at 60 °C for 2 days. Sections were photographed in a field emission gun transmission electron microscopy (TEM, Tecnai G2 TF20 Super TWIN, FEI company, Hillsboro, OR, USA).

Formvar carbon-coated grids were loaded with a drop of prepared VLDL and excess solution wicked away by a tissue prior to staining with 1 % sodium phosphotungstate solution (pH 5.9) for 40 s. Ultrastructure analysis of isolated VLDL was conducted by electron micrograph with a thermal field emission scanning electron microscope (FE-SEM)(JEOL JSM-7800F, Tokyo, Japan).

### Western blotting

Proteins were extracted from tissue homogenates (devoid of yolk), cell lysates, and isolated VLDL using a standard RIPA buffer containing a protease and phosphatase inhibitor cocktail (Cat. No. 78425, Thermo Fisher Scientific). Proteins were separated using SDS-PAGE (Mini-gel system, Bio-Rad, Hercules, CA, USA). After transfer, the membranes were blocked and probed with the primary antibody against chicken MTTP-M, ApoB, or ApoVLDL-II as described previously using a horseradish peroxidase (HRP)–conjugated secondary antibody against rabbit or mouse IgG (Cell Signaling Technology) and enhanced chemiluminescence (Pan et al., 2012).

### RT-PCR analysis

Freshly collected ovarian follicles and stroma were quickly dissected on ice into 1 mm^3^ pieces. Tissue pieces and isolated cells were immersed in RNAlater^TM^ (Invitrogen, Waltham, MA) and stored at –80 °C until used. Total RNA extraction, and random priming reverse transcription were conducted as described previously using commercial kits (Applied Biosystems, Waltham, MA)(Pan et al., 2012). The sequence of primer pairs as; F: 5′-TTCCAGCTTCCACGTATCCC-3′ and R: 5′-ATTTGGACGTTGCTTGAGCTG-3′ for ApoB (GenBank accession number: NM_001044633, 181 bps in amplicon)(Cheng et al., 2018a), and 5′-GCTAGCCTTTTCCAGCTAC-3′; 5′-ATTTTGGCACCTGTTTTTCG-3′ for MTTP-M (GenBank accession number: KC176805, 292 bps in amplicon) were used for PCR amplification using a commercial kit (TaKaRa Bio Inc., Shiga, Japan). The PCR cycle was set as template denaturation at 95 °C for 30 s, primer annealing at 60 °C for 30 sec, and extension at 72 °C for 1 min.

### MTTP activity analysis

MTTP activity measurements were made using samples prepared and assayed according to the instructions enclosed in the commercial kit (Cat. No. MAK110, Sigma-Aldrich). For measurements with whole tissue samples, yolk-free follicle pieces and muscle were first minced, homogenized, and suspended in a homogenization buffer containing 100 mM Tris-HCl, 1.5 M NaCl, 10 mM EDTA, 0.5 mM PMSF, 20 μg/mL leupeptin. The suspension was then sonicated on ice with six 10-second bursts (550 W). For both freshly isolated follicular cells and cells after cultures, cells were pelleted and suspended in a buffer containing 10 mM Tris, 150 mM NaCl, 1 mM EDTA, 0.5 mM PMSF, and 50 μg/mL leupeptin, and sonicated on ice with six 5-second bursts (550 W). Protein concentration of the homogenates was adjusted to 10 mg/mL. In each well of fluorescence-based microplates (Cat. No. M33089, Thermo Fisher Scientific), 182 µL assay buffer, 4 µL donor and 4 µL acceptor particle, and 100 µg of protein homogenate were combined and allowed to incubate at 37 °C. Triplicate wells were prepared for each tissue or cell type and treatment. The fluorescence (Ex/Em: 465/535 nm) of prepared samples was continuously recorded for up to 150 min. MTTP activity was expressed as percentage of lipid transfer as calculated by the following equation: percentage of lipid transfer = (arbitrary fluorescence units in assay wells − blank values)/(total fluorescence units − blank values) × 100.

### Metabolic labeling

The amino acid analog, azidohomoalanine (Click-iT™ AHA, Thermo Fisher Scientific) was used in metabolic labeling studies to trace ApoB synthesis and secretion. Medium from cells previously incubated overnight with AHA (50 μM) was collected and used to isolate VLDL. Newly synthesized proteins possess AHA and are labeled with biotin using the Click-iT® Protein Reaction Buffer kit and Biotin Azide (Thermo Fisher Scientific). Proteins are separated by SDS-PAGE, transferred onto membranes, probed by streptavidin-HRP conjugate, imaged using enhanced chemiluminescence photographed per a standard Western blot procedure. Viability of cell cultures used in all studies were determined by commercial CCK-8 kits (ab228554, Abcam, Cambridge, UK).

### ApoB quantification by ELISA

Culture medium from heat-stressed and control cells was collected for VLDL isolation and for ApoB quantification using a chicken-specific ELISA kit (No. CSB-EL001918CH, Cusabio, Houston, TX, USA). Total protein content was determined by Bradford assay with a commercial kit (ab102535, Abcam).

### Statistics

Data was analyzed by one-way ANOVA followed by Student's t-test or Tukey multiple comparison tests. Results were expressed as means ± SEM. Mean differences were considered significant at *P* < 0.05. Statistical analysis was performed using SPSS for Windows 13.0.

## Results

### MTTP and ApoB expression in ovarian tissues of chickens

**Fig. 1:**
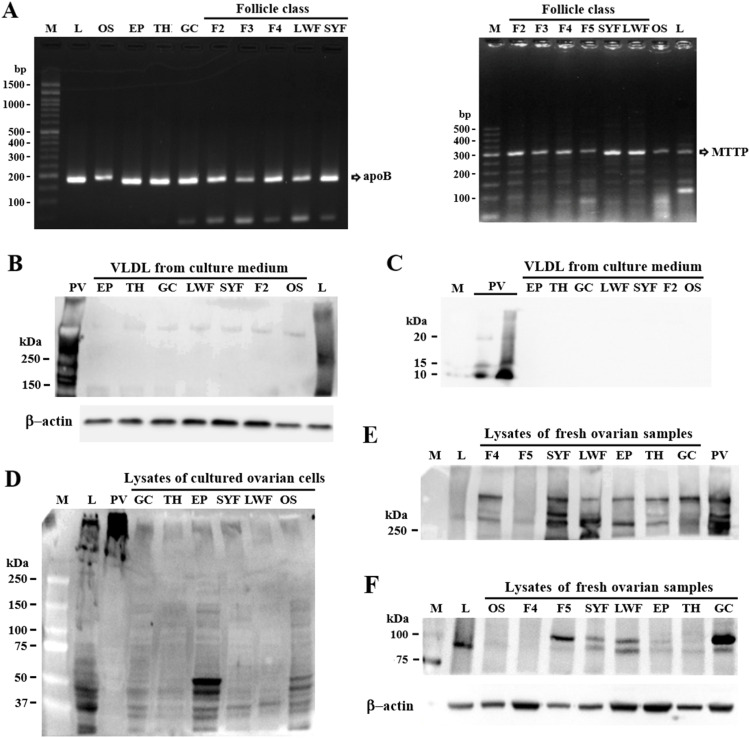
Expression of ApoB and MTTP in chicken ovarian tissues and their secretion of ApoB. Total RNA extracts from ovarian tissues including stroma (OS), hierarchical follicles (F2-F5), small yellow follicles (SYFs), large white follicles (LWFs), freshly isolated granulosa (GC), theca (TH), and epithelial (EP) cells (from F2-F5 follicles), and liver (L) from a laying hen were used to amplify ApoB and the M subunit of MTTP (MTTP-M) transcripts by RT-PCR (panel A). After seeding, EP, TH, and GC cells (from F3 and F4 follicles) and total cells from LWF, SYF, OS, and F2 follicles at 85 % confluence were cultured overnight prior to culture medium harvest for VLDL isolation. The d < 1.006 g/ cm^3^ fraction of harvested medium and cells were used for ApoB (panel B and D, respectively) and ApoVLDL-II (panel C) analysis by Western blot at equal total protein. Amounts of ApoB and MTTP-M in freshly isolated EP, TH, and GC cells (from F2 and F3 follicles) and ovarian tissues were measured by Western blot (panel E and F, respectively). Laying hens’ plasma VLDL (PV) and liver (L) served as references. M; marker.

Both ApoB and MTTP-M transcripts were detected in various classes of ovarian follicles including SYFs, LWFs, and hierarchical follicles (F2 to F5), as well as in the ovarian stroma (OS) and follicle GC, TH, and EP cells (Fig. 1 panel A) using RT-PCR. Western blot studies with VLDL isolated from culture medium confirmed ApoB secretion by follicle cells and that secreted VLDL did not contain detectable ApoVLDL-II with an equal amount of protein loaded (Fig. 1 panel B and C). In isolated cell culture preparations, the profile of ApoB fragments below 250 kD resembled those from laying hen livers but lacked ApoB fragments above 250 kDa. However, homogenates of freshly isolated follicle cells and ovarian tissues showed several ApoB fragments above 250 kDa similar to those of plasma VLDL (Fig. 1 panel D and E), suggesting that degradation occurred during the culture procedure despite addition of proteolysis inhibitors. Panel C shows the lack of ApoVLDL-II that would be expected if ApoB in the medium resulted from the release of VLDLy contained within the tissue. These results argue against VLDL/VLDLy internalized from the circulation as responsible for the ApoB-reactive bands observed in the isolated follicle cells after culturing. In addition to the liver, intestine, and kidney that have been previously shown to synthesize ApoB for VLDL assembly in chickens (Fig. S2 panel B)(Griffin et al., 1982; Kirchgessner et al., 1987; Tarugi et al., 1990; Walzem et al., 1999), for the first time, a remarkable level of MTTP-M in 2 isoforms was detected in various classes of ovarian follicles and follicular GC, TH, and EP cells (Fig. 1 panel F).

**Fig. 2:**
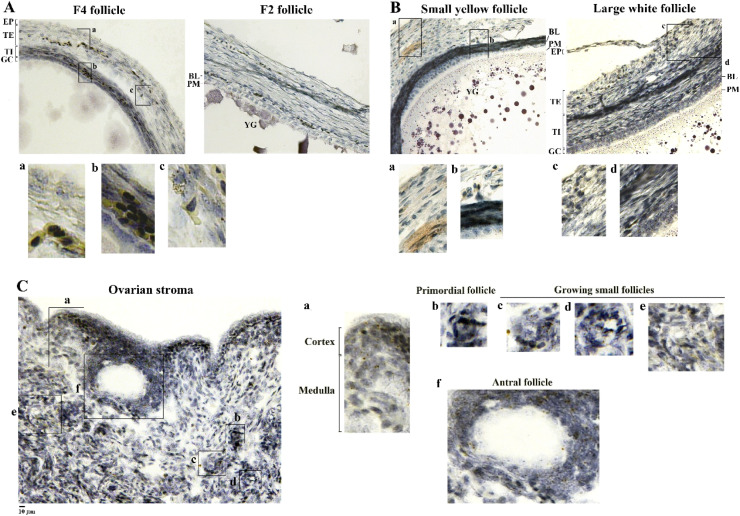
In situation hybridization of ApoB mRNA in chicken ovarian tissues. Ovarian follicles (panel A and B) and stroma (panel C) were used for in situ hybridization of ApoB mRNA using a commercial kit with a specific target probe. In panel C, ovarian cortex and medulla, and various classes of stromal small follicles were noted for ApoB expression. Scale bars; 10 μm, magnification 20 × . EP; epithelial layer; TE; theca externa, TI; theca interna, GC; granulosa layer, PM; plasma membrane (oolemma), BL; basal lamina, YG; yolk granules. .

In situ hybridization specified ApoB transcripts in the stroma and follicle tissues with the greatest amount in TH layers followed by EP and GC cells in hierarchical follicles, SYFs and LWFs (Fig. 2 panel A and B**)**. Within the stroma, the cortical regions had higher amounts of ApoB transcripts than the medulla, and stromal cells. ApoB transcripts were also found in small growing follicles from the primordial to antral stage (Fig. 2 panel C).

**Fig. 3:**
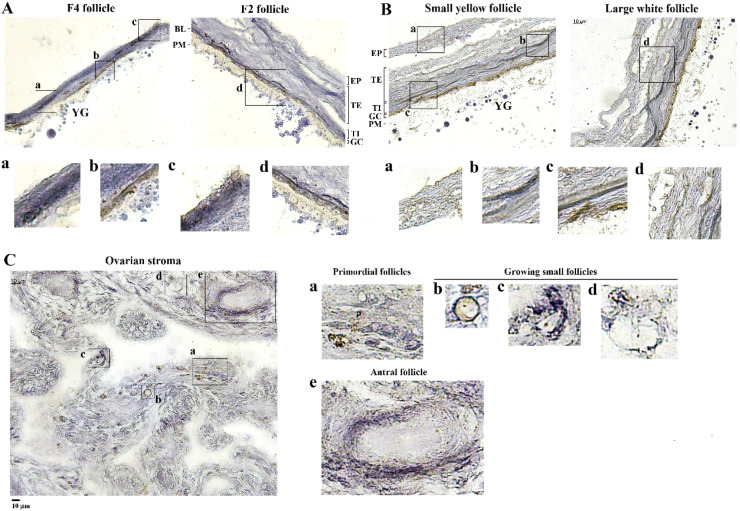
Immunohistochemical analysis of MTTP expression in chicken ovarian tissues. Ovarian follicles (panel A and B) and stroma (panel C) were used for immunohistochemistry using an antibody raised against the synthetic epitope of chicken MTTP-M subunit. In panel C, various classes of stromal small follicles were noted for MTTP-M expression. Scale bars; 10 μm, magnification 20 × . EP; epithelial layer, TE; theca externa, TI; theca interna, GC; granulosa layer, PM; plasma membrane (oolemma), BL; basal lamina, YG; yolk granules.

Immunohistochemical analysis confirmed MTTP protein expression in both ovarian stroma and follicle tissues, with higher abundances in the GC layers of SYFs, LWF and hierarchical follicles, while considerable MTTP amounts were also observed in the TH and EP layers of SYFs, but only a trace amount was observed in the hierarchical follicles (Fig. 3 panel A and B). Both stromal cells and small growing follicles from primordial to antral follicles were also identified as processing MTTP protein (Fig. 3 panel C).

### Functional assessments of ovarian MTTP and ApoB expression in chickens

**Fig. 4:**
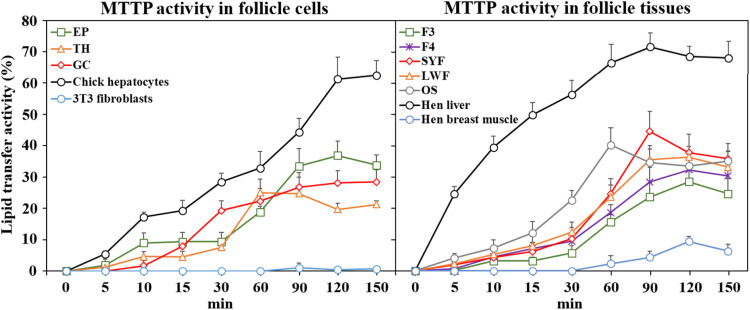
MTTP activity in chicken ovarian follicles and cells. Freshly isolated epithelial (EP), theca (TH), and granulosa cells (GC) from ovarian F2 and F5 follicles and whole tissues of F3, F4, small yellow follicles (SYFs), large white follicles (LWFs), and stroma (OS) were used for MTTP activity analysis (*n* = 3 hens). Hens’ breast muscle and mouse 3T3 fibroblasts served as negative references, while hens’ livers and isolated chick hepatocytes were used as a positive control.

**Fig. 5:**
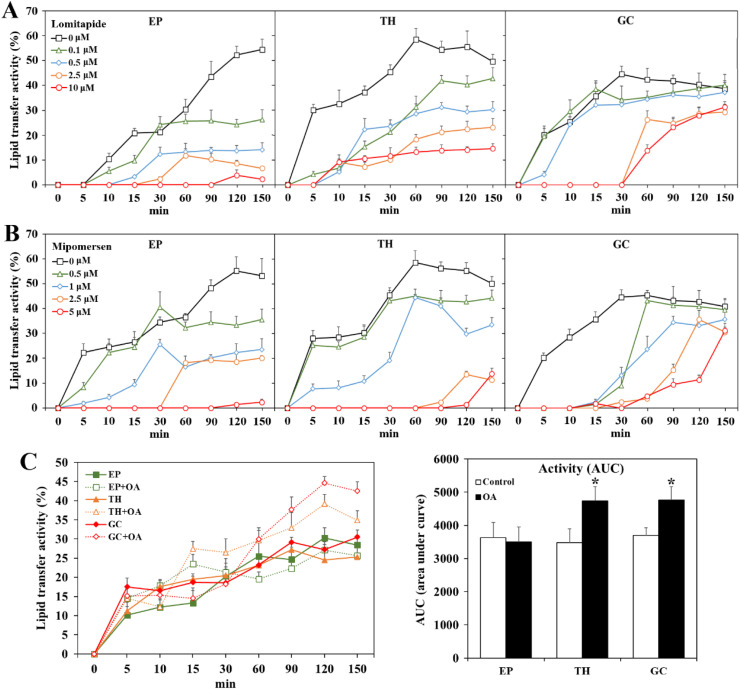
MTTP activity of chicken ovarian follicle cells respond to Lomitapide and Mipomersen inhibition and oleate stimulation. Ovarian granulosa (GC), theca (TH), and epithelial (EP) cells (from F2-F4 follicles) grown to 85 % confluence were treated with Lomitapide (Lom) or Mipomersen (Mip) (a MTTP and ApoB inhibitor, respectively, panel A and B) at indicated concentrations, or their combination (2.5 μM for each, panel C) for 2 hr and replaced with new medium in the presence or absence of oleate (OA, 0.1 mM) for overnight culture. Cells were thereafter harvested for MTTP activity analysis. *; significant difference (*P* < 0.05, vs. corresponding control, *n* = 3).

Within the ovary, the highest activity of MTTP was found in SWF and LWF while hierarchical F4 and F3 follicles had lower lipid transfer activity (Fig. 4). Among the different follicular cell types, GC and EP cells had greater lipid transfer activity than TH cells. Hen's liver and primary chick hepatocytes possessed a dramatically higher MTTP activity than any ovarian tissue or cell type. Both muscle and mouse 3T3 fibroblasts possessed barely detectable lipid-transfer activity. The MTTP activity of chicken follicle cells responded to pharmacological inhibition by the MTTP inhibitor Lomitapide (Wetterau et al., 1998) and surprisingly by the ApoB antisense oligonucleotide Mipomersen in a dose-dependent manner (Fig. 5 panel A and B). Isolated TH and GC cells also showed increased lipid transfer activity in the presence of supplemental OA (*P* < 0.05, Fig. 5 panel C).

Mipomersen, a 2nd generation of antisense oligonucleotide (ASO) with chemical modifications to form a stable single-strand chimeric in 20-bp length, specifically targets apoB-100 mRNA to inhibit apoB synthesis (Lee et al., 2013b). Studies have confirmed consistent pharmacokinetic properties of Mipomersen across species including humans, monkeys, mouse, and rats (Geary et al., 2015). Both Lomitapide and Mipomersen have been approved by the U.S. Food and Drug Administration (FDA) and clinically a combination of the two drugs can potentiate the reduction of LDL-cholesterol (Rader and Kastelein, 2014). Lomitapide has been shown to induce autophagic death in cancer cells (Lee et al., 2022), whereas doses of Lomitapide and Mipomersen used in this study had no significant effects on cell viability except Lomitapide at a level > 2.5 μM (Fig. S3).

To justify the crossover reactivity of Mipomersen interference in chicken species, the sequence of Mipomersen was blasted to the reported chicken (*Gallus gallus*) and human (*homo sapiens*) apoB mRNA sequences (access #: NM_001044633.1 and NM_000384.3 in Genbank, respectively). Results showed 3 identical segments at 8, 7, and 5-bp length matched to the sequence of chicken and human apoB mRNA **(**Fig. S4**)**.

**Fig. 6:**
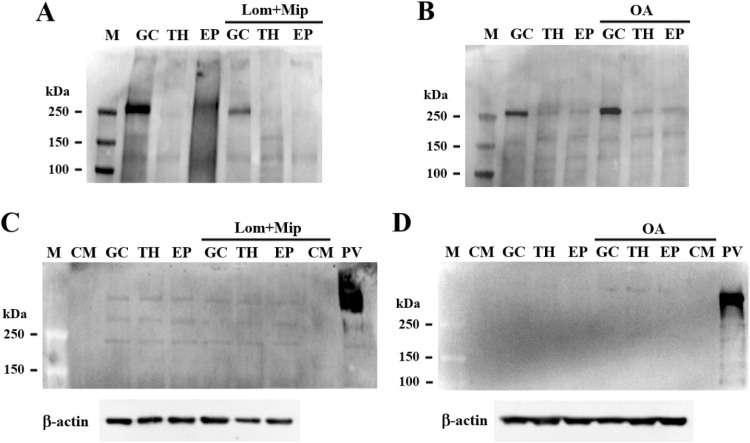
Metabolic labeling analysis of ApoB secretion by ovarian follicle cells. Ovarian granulosa (GC), theca (TH), and epithelial (EP) cells (from F2-F4 follicles) at 85 % confluence were treated with Lomitapide and Mipomersen (Lom and Mip, a MTTP and ApoB inhibitor, respectively, 2.5 μM) for 2 hr and replaced with new medium containing azidohomoalanine (AHA, 50 μM) in the presence or absence of oleate (OA, 0.1 mM) for overnight culture. Culture medium was collected for VLDL isolation. The newly synthesized proteins identified by AHA incorporation in VLDL extracts were labeled with biotin azide and analyzed by the regular Western blot method using streptavidin-HRP conjugate as a probe (panel A and B). The secretion of ApoB following treatment with Lom and Mip inhibitors or OA stimulation was validated by Western blot analysis using an antibody raised against chicken ApoB (panel C and D). The protein extracts of laying hens’ plasma VLDL (PV) and cell-free culture medium (CM) after ultracentrifugation against the density buffer at 1.006 g/cm^3^ served as references. M; marker.

**Fig. 7:**
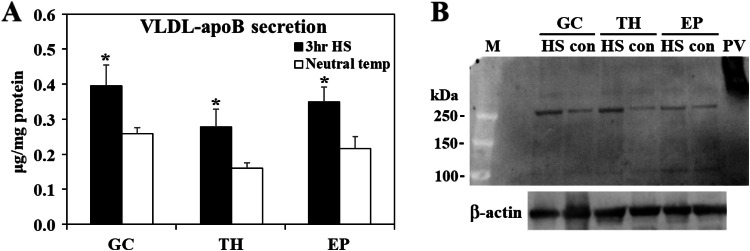
Heat stress induces ApoB secretion from ovarian follicle cells. Ovarian granulosa (GC), theca (TH), and epithelial (EP) cells (from F2-F4 follicles) were grown at 37 °C to 85 % confluence were subjected to heat stress (HS) by incubation at 42 °C for 3 hr and then returned to 37 °C for overnight culture. Control cultures were maintained at constant 37 °C for the same incubation time. In the next day, culture medium was collected for VLDL isolation and collected VLDL was used for ApoB quantification using a commercial ELISA kit (panel A). Western blot analysis with an antibody raised against chicken ApoB was used to validate ApoB presence (panel B). The protein extracts of laying hens’ plasma VLDL (PV) served as a reference. M; marker.

To confirm de novo assembly and secretion of apoB-LP, cells were treated with L-azidohomoalaine, an amino acid analog of methionine. Harvested media was fractionated by ultracentrifugation to produce a d < 1.006 g/mL fraction that contained newly assembled apoB-LP, i.e. VLDL. The VLDL-apoB assembly and secretion process in these cell types was inhibited by Lomitapide and Mipomersen and stimulated by OA addition (Fig. 6 panel A to D). The use of L-azidohomoalaine to trace *de novo* synthesis and secretion of ApoB may increase the susceptibility of degradation leading to fragments mainly around 250 kDa (Fig. 6 panel A and B). As observed in heat-stressed hens (Cheng et al., 2018a; 2018b), cultured follicular cells showed increased VLDL-ApoB secretion following a rapid increase in environmental temperatures (*P* < 0.05, Fig. 7).

**Fig. 8:**
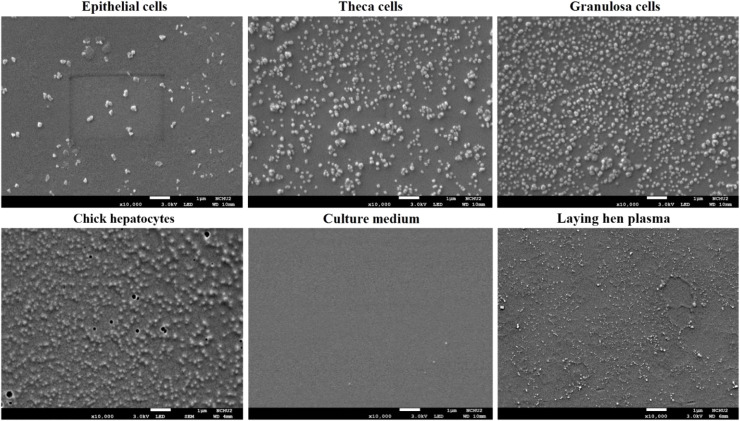
Ultrastructure analysis of secreted VLDL by follicle cells. Ovarian granulosa, theca, and epithelial cells (from F2-F4 follicles) grown to 85 % confluence were cultured overnight prior to culture medium harvest for VLDL isolation. The d < 1.006 g/ cm^3^ fraction of harvested medium was imaged using scanning electron microscopy (SEM). VLDL isolated from laying hens’ plasma and medium harvested from chick hepatocyte cultures served as positive controls. Cell-free medium served as a negative reference. Scale bars; 1 μm, magnification;10,000 × .

The culture medium of follicle EP, TH, GC cells were then used as sources for VLDL isolation and visualization by SEM. All the three types of cells were able to secrete VLDL that were morphologically identical to those secreted by chick hepatocytes (Fig. 8). In contrast to the narrower 30-35 nm distribution characteristic of VLDLy found in hen plasma during egg production (Griffin et al., 1982; Walzem et al., 1994; MacLachlan et al., 1996), the VLDL particles secreted by cultured follicular cells had diameters of 50-200 nm, while those by chick hepatocytes ranged approximately from 50 to 150 nm (Fig. 8).

### Ultrastructural analysis of VLDL assembly in ovarian follicle cells

**Fig. 9:**
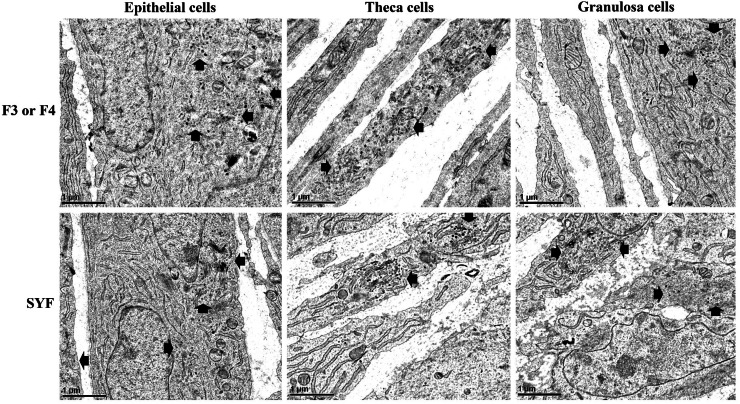
Electron micrograph of chicken ovarian follicles. Ovarian follicles were fixed and stained by imidazole-buffered osmium tetroxide procedure and imaged using transmission electron microscopy (TEM). Scattered VLDL (black to dark grey particles indicated by arrows) were observed in epithelial, theca, and granulosa cells of the hierarchical (F3 or F4) and small yellow follicles (SYFs). Scale bars; 1 μm, magnification; 2,550 × .

**Fig. 10:**
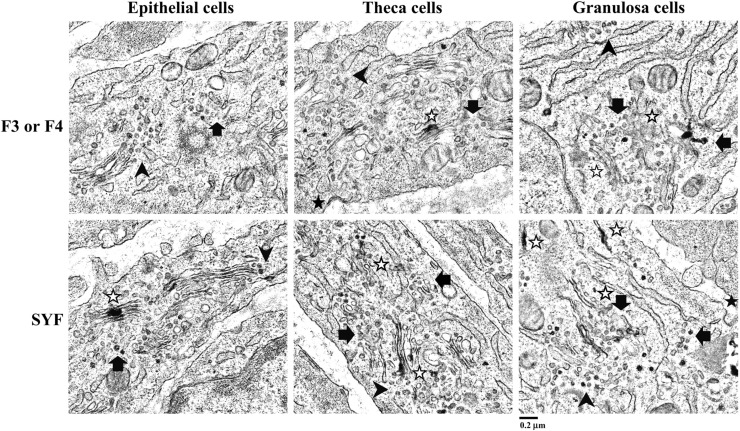
Analysis of VLDL assembly and traffic in chicken ovarian follicle cells. Ovarian follicles were fixed and stained by imidazole-buffered osmium tetroxide procedure and imaged using transmission electron microscopy (TEM). VLDL particles with diameters around 50-60 nm were observed in epithelial, theca, and granulosa cells of the hierarchical (F3 or F4) and small yellow follicles (SYFs). Images were noted with VLDL trafficking from the ER to GI (black arrows), GI toward cell surface (black arrow heads), VLDL accumulation within ER or GI (blank stars), and coated pits (black stars). Scale bars; 0.2 μm, magnification; 7,000 × . ER; endoplasmic reticulum. GI; Golgi apparatus.

**Fig. 11:**
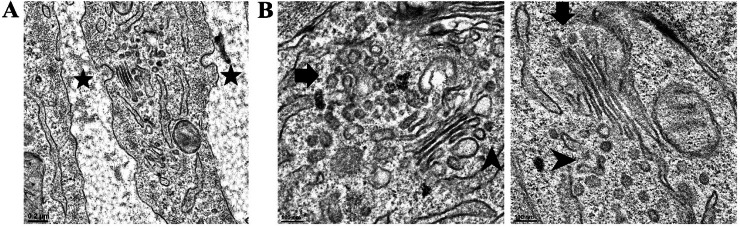
Coated pits and VLDL transport vesicles in chicken ovarian follicle cells. Ovarian follicles were fixed and stained by imidazole-buffered osmium tetroxide procedure and imaged using transmission electron microscopy (TEM). Very few VLDL were observed in the interstitial spaces. Coated pits on the cell surfaces were notably devoid of VLDL clustering in the pocket (black stars, panel A). VLDL were trafficked in secretory vesicles; mostly with single particle per vesicle, departing from ER to GI (black arrows) and from GI toward cell membranes (black arrow heads). Scale bars; 0.2 μm and 100 nm and magnification; 7,000 × and 19,500 × for panel A and B, respectively. ER; endoplasmic reticulum. GI; Golgi apparatus.

Ultrastructural examination of EP, TH, and GC cells in both hierarchical follicles and SYFs revealed VLDL-like particles (dark gray to black particles, Fig. 9). Sections manifested lipoprotein particles trafficking from ER (endoplasmic reticulum) to accumulate within the Golgi apparatus (black arrows) prior to movement from the Golgi apparatus towards the plasma membranes for secretion (black arrow heads). VLDL intermediates were found to accumulate within the ER (blank stars), while assembled VLDL accumulated within the or Golgi apparatus (blank stars) (Fig. 10). Coated pits observed on the cell surfaces were devoid of VLDL clustering in the pocket and very few VLDL were present around plasma membranes (black stars, Fig. 10, Fig. 11 panel A). Interestingly, the traffic of VLDL within chicken follicle cells was carried out mostly in a one particle per transport vesicle-dependent manner (Fig. 11 panel B).

## Discussion

We previously found that both ApoB transcripts and proteins were present in the ovarian tissues of laying hens (Cheng et al., 2018a; 2018b). The expression of ApoB and MTTP genes and presence of those proteins in hens’ ovaries are now shown to be key parts of an active VLDL assembly process; albeit the assembly processes as assessed by MTTP activity were less active than those of hepatocytes. VLDL were recovered from the d < 1.0063 g/mL fraction of media recovered from cultures of follicular GC, TH and EP cells were sufficiently abundant for visualization by SEM. The particles recovered had diameters of 50-200 nm, even larger than those secreted by chick hepatocytes. The VLDL secreted by EP, TH and GC did not contain Apo-VLDL-II further supporting the conclusion that these cells secrete larger generic VLDL not VLDLy.

The activity of MTTP was similar in basal cultures of the three types of follicular cells, but only GC and TH responded to OA addition. Yang et al. (2019) reported that the glycerophosphate pathway of TAG synthesis is present in chicken ovary but did not separate cell types within ovarian tissue. It is possible a variation in TAG synthesis capacity among EP, TH and GC underlying the difference in OA responsiveness. The VLDL assembly and secretion in follicular cells showed expected responses to Lomitapide and Mipomersen inhibition when added to cell cultures. The somewhat unexpected finding that Mipomersen can suppress MTTP activity suggests that ApoB protein abundance in the microsomal preparations can affect the *in vitro* assessment of lipid transfer activity. This suggestion is supported by the mutually stabilizing effects that exist between ApoB and MTTP (Leung et al., 2000). Overall, the findings support the possibility that VLDL assembly processes in the avian ovary may act on cellular lipid homeostasis to protect follicular cells against stress and sustain follicle development (Cheng et al., 2018a; 2018b; Wei et al., 2019).

As noted in the introduction, estrogen stimulation of the liver in laying hens greatly promotes and alters hepatic VLDL assembly and secretion to create the distinctive VLDL biology required to deliver TAG to the ovary for yolk deposition and following embryonic use. The distinctions between generic VLDL and VLDLy include smaller diameter, a higher fraction of TAG and resistance to lipoprotein lipase (Griffin et al., 1982; Nimpf et al., 1988; Schneider et al., 1990; Walzem et al., 1999; Boyle-Roden and Walzem, 2005). Approximately, 30 % radioactivity was recovered in the rapidly growing ovarian follicles of laying hens killed 6 h after intravenous injection with radio-labeled laying hen VLDL (presumably VLDLy)(Bacon et al., 1978). Early studies found that the basal lamina surrounding the granulosa layer exterior to the oolemma functions as a mechanical and electrostatic barrier to exclude larger VLDL particles (>50 nm) and allows suitable VLDLy particles (30-35 nm) to pass through to access the oolemma where VLDLy are internalized for yolk formation through receptor-mediated endocytosis (Evans et al., 1979; Perry and Gilbert, 1979). Laying hen follicles exhibited 100-fold more VLDLy particles bound on the oolemma surfaces and 10-fold more membrane areas covered by coated pit regions than those on mammalian fibroblasts (Perry and Gilbert, 1979). The ligand recognition was further shown residing in the carboxyl terminal of ApoB-100 of laying hen VLDL not through apoVLDL-II (Nimpf et al., 1988; Bujo et al., 1995). Very few VLDL were observed to associate on the follicle cell surfaces and the coated pits were devoid of VLDL clustering, while by contrast the follicles engaged in yolk deposition showed massive VLDL adherence to the oolemma surfaces prior to uptake through endocytosis (Perry and Gilbert, 1979). The present study found trace VLDL in the interstitial space and pockets of coated pits lacked VLDL clustering consistent with these early reports. However, VLDL with particle size around 50-60 nm were found intracellularly and appeared to traffic in orderly progression toward secretion in the follicular cells. These results argue against the intracellular VLDL observed in the transmission electron microscopy (TEM) studies being present due to internalization of circulating VLDLy and supports instead that these particles were synthesized locally by the follicular cells. The observations are consistent with the findings arising from the metabolic labeling studies of ApoB and further confirm the presence of functional VLDL assembly and secretion by cultured follicle cells t o produce generic VLDL with diameters of 50-200 nm. While the purpose of VLDL assembly in the livers of laying hens is dedicated to yolk formation, the functional assembly process within the ovary is less apparent but may regulate lipid content and composition in follicular cells and yolks to support reproduction.

Functional MTTP and ApoB have been reported in human ovarian granulosa cells in conjunction with functional VLDL assembly and secretion (Gautier et al., 2010). The ApoB concentrations in follicular fluid were positively correlated with better embryo quality and a higher pregnancy rate in patients with IVF (*in vitro* fertilization) or ISCI (intracytoplasmic sperm injection)(Gautier et al., 2010); findings consistent with a protective action for VLDL assembly. Further, in mammals, the UPR (unfolded protein response) signaling in granulosa cells is associated with follicle development and luteinizing process, in which the levels of XBP1 (X-box binding protein 1) mRNA in cumulus cells of the oocyte that achieves fertilization are higher than those incompetent for fertilization (Huang et al., 2017). Metabolomic analysis found dysfunctional metabolism of follicular glycerolipids and sphingolipids in individuals experiencing infertility due to polycystic ovary syndrome (PCOS) as indicated by lower levels of TAG, phospholipids, sphingolipids, and glycosphingolipids in the follicular fluid, but increased abundance of lysoPE (lysophosphatidylethanolamine) (Liu et al., 2019). These changes were highly related to the lower fertilization rate and poor embryo characteristics for IVF procedures. Recently, sphingolipids were suggested to participate in UPR signaling and ER stress to regulate cell fate (Park, and Park, 2020). Accordingly, these results highlight the importance of cargo lipid export by ApoB-LP in ovarian tissues to maintain cellular lipid homeostasis and protect the cells under stress conditions such as heat and excessive nutrient influx.

We identified disordered lipid metabolism in hens with failing/failed egg production due to overfeeding (Walzem et al., 1994). Overfed hens experiencing failure of yolk deposition and induced ovarian involution exhibited an increase of VLDL diameter indicative of loss of yolk-targeting in conjunction with the appearance of a unique HDL subpopulation. These ovarian dysfunctions were reproducible in a separate strain of chickens with the further finding of lipotoxic changes (Pan et al., 2012). We subsequently demonstrated that overfeeding hens exhibited follicular inflammation and lipotoxicity due to ceramide and TAG accumulation, which led to impaired ovarian functions and even provoked follicular atresia (Pan et al., 2012; 2014; Xi et al., 2012; Liu et al., 2014). An RNA-seq analysis by others reported differential expression of ovarian ApoB and MTTP genes functioning in the context of excessive lipid transport in overfed hens (Wei et al., 2019). Since VLDLy is massively targeted to the ovary for yolk deposition, it is reasonable to suppose that follicular tissues are satiated with available lipids and thus at a risk for lipid overload. Particularly in hens provided free access to feed when VLDLy may be saturated with NEFA (Leclercq et al., 1979). Additionally, laying hen granulosa cells were reported to possess LPL activities up to half of that of adipose (Benson et al., 1975). The presence of VLDL assembly and secretion in chicken ovaries therefore may be evolutionarily conserved and serve as a mechanism to export excess lipids from follicular cells to protect the cells against lipotoxicity as suggested for the failing human heart (Borén et al., 1998). Alternatively, the ability to reduce NEFA delivery into yolk by removal from VLDLy prior to uptake may control yolk quality for proper nutrition for the embryo.

Within the ovary, follicle development starts with a recruitment of a set of small follicles to grow and the numbers of follicles diminish daily as retained follicles grow in size to become SYFs (Johnson, 2000). Once the F1 is ovulated, one SYF is recruited to advance to the final yolk-filled hierarchical stage and committed to ovulation following further growth. Our previous studies using microarray and proteomic approaches found that differential expression of ApoB gene in the SYFs of hens under thermal stress (Cheng et al., 2018a; 2018b). The present study further confirmed that cultured follicle cells respond to transient heat stress with increased VLDL-apoB secretion. Since heat stress can disrupt egg laying and increases lipid accumulation in the peripheral tissues of chickens (Brugaletta et al., 2022), ApoB expression within SYF thus may function as a control process to maintain follicle quality. Other proteomic studies showed that differential abundance of ovarian follicle ApoB was involved in the selection of SYF for entry into the hierarchy (Chen et al., 2020; Ghanem et al., 2021). ApoB-LP assembly and secretion within the developing yolky follicles may play a crucial role in the protection of follicles to ensure normal development as well as an appropriate composition of lipids deposited into yolk for embryo use.

In summary, chicken ovarian tissues possess functional MTTP and apoB capable of VLDL assembly and secretion. The process responds to heat stress and OA stimulation and may act as a protective mechanism against fuel and physical stress to secure follicle development and/or nutritional quality control of yolk for embryo development.

## Disclosures

The authors declare no conflicts of interest.

## Declaration of competing interest

The authors declare no conflict of interest.

## Appendix Supplementary materials

Image, application 1
