# Supplementary material for: Ovarian expression of functional MTTP and apoB for VLDL assembly and secretion in chickens
Source: Poult Sci. 2025 Mar 6;104(5):104993. doi: 10.1016/j.psj.2025.104993 (PMC11951013; doi:10.1016/j.psj.2025.104993)
Supplement: Supplementary file 1 [file mmc1.docx]

**Supplementary results**

**
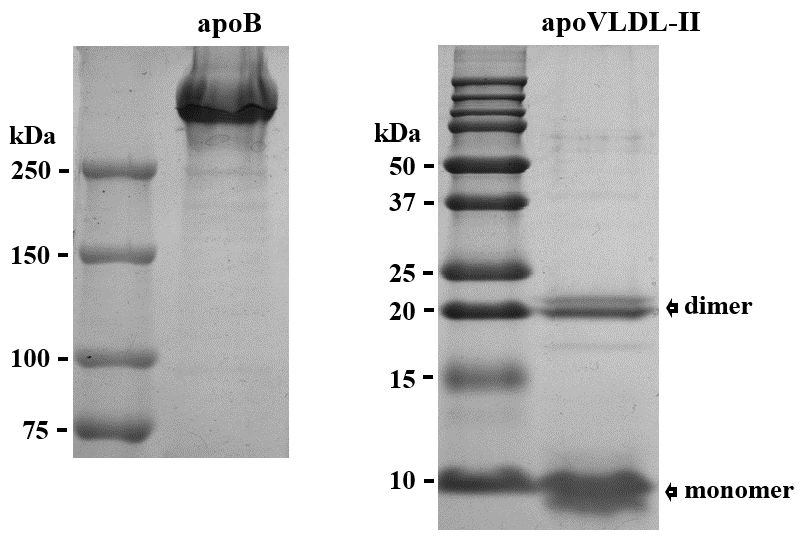
**

**Supplementary Figure S1. Purification of apoB and apoVLDL-II from plasma VLDL of laying hens**

Purified apoB and VLDL-II from the plasma VLDL of laying hens were used as antigens to immunize mice and rabbits, respectively.


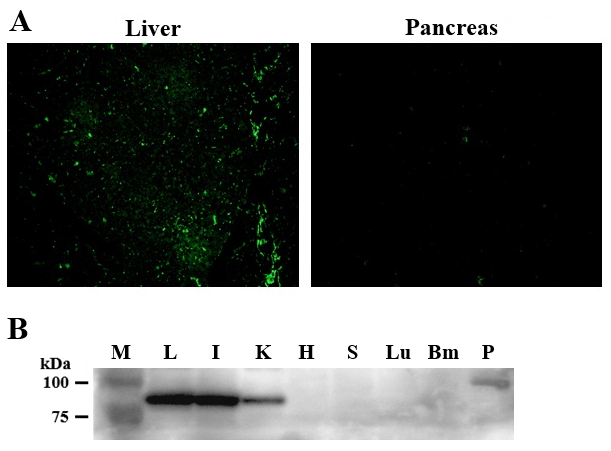


**Supplementary Figure S2. Specificity validation of the antibody raised against chicken MTTP-M.**

A synthetic peptide (CRKVFSTASDSSGSWF) corresponding to the carboxy-terminal of the M subunit of chicken MTTP was used as an epitope to raise antibody in rabbits. The antisera were purified by affinity chromatography through a protein A-sepharose column to obtain IgG fraction. The liver (L), intestine (I), kidney (K), heart (H), spleen (S), lung (Lu), breast muscle (Bm), and pancreas (P) from a laying hen were used for immunohistochemistry (panel A) or Western blot analysis (panel B) to validate the antibody specificity.


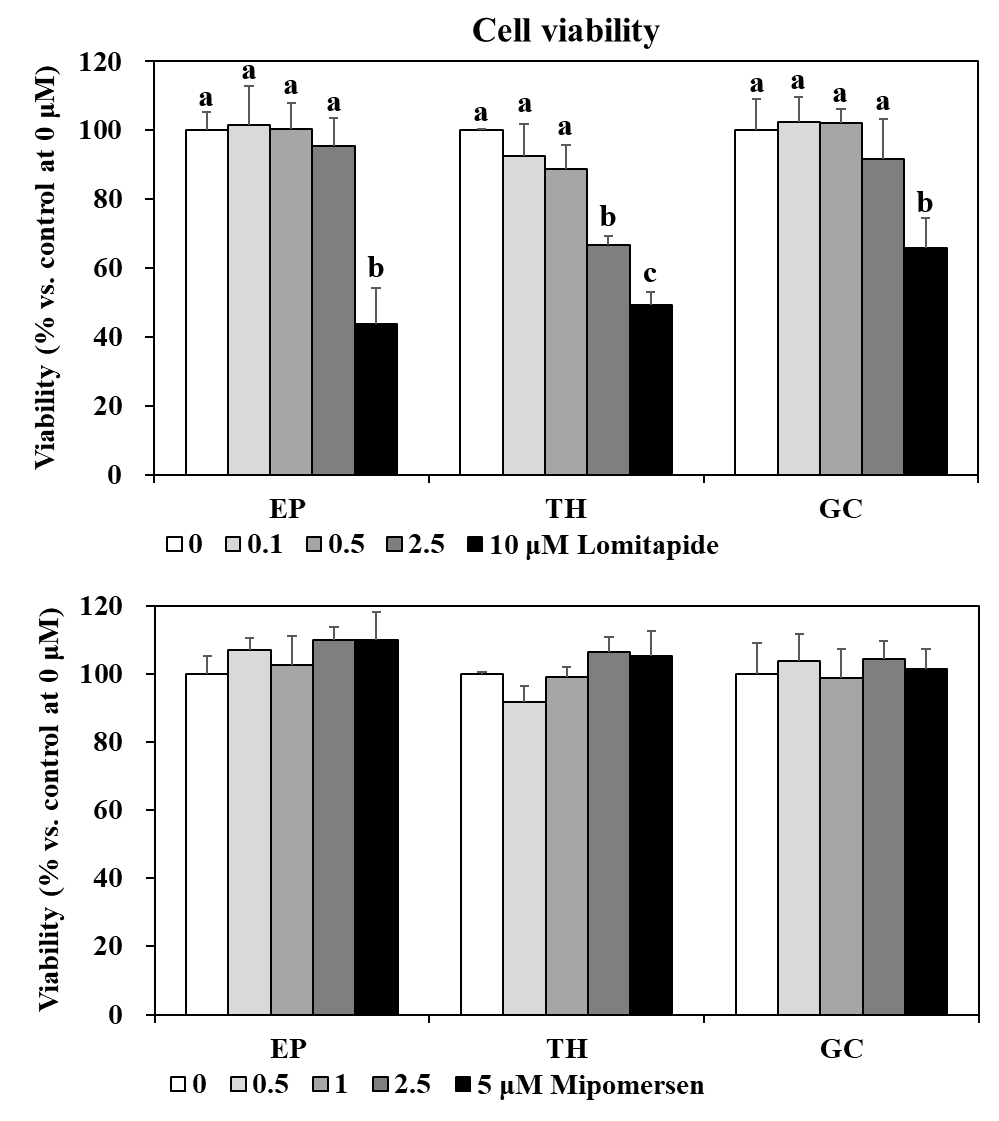


**Supplementary Figure S3.** **Effects of Lomitapide and Mipomersen on follicle cell viability.**

Ovarian granulosa (GC), theca (TH), and epithelial (EP) cells (from F2-F4 follicles) grown to reach 85% confluence were treated with Lomitapide or Mipomersen (a MTTP and apoB inhibitor, respectively, panel A and B) at indicated concentrations for 2 hr and replaced with new medium for overnight culture. Cells were then harvested for viability analysis. Means with different letters within the same cell type are significantly different (P<0.05, n=4).


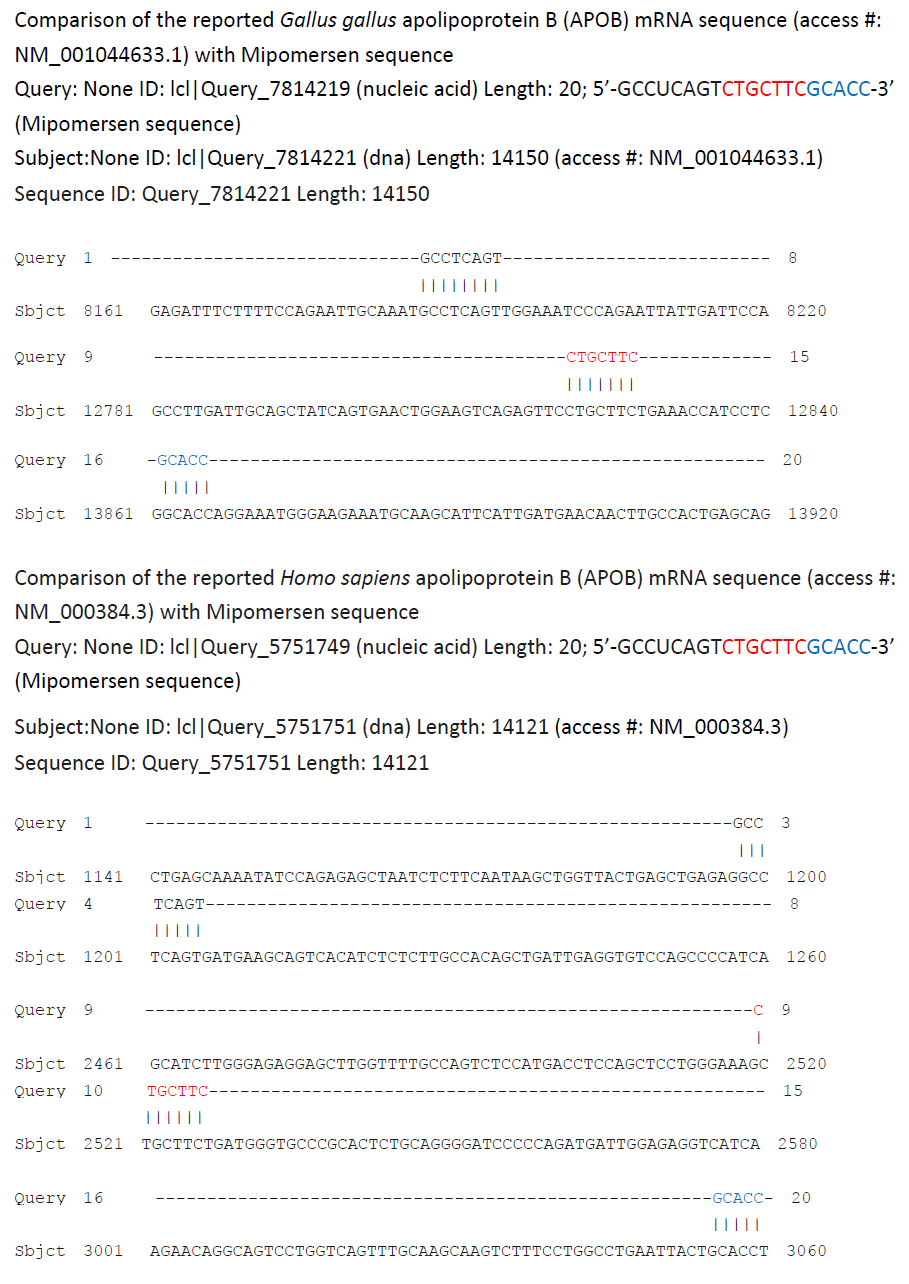


**Supplementary Figure S4.** **Comparison** **of Mipomersen sequence to chicken and human apoB mRNA sequence.**

The 20-bp Mipomersen (5’-GCCUCAGTCTGCTTCGCACC-3’) was blasted to the reported chicken (*Gallus gallus*) and human (*homo sapiens*) apoB mRNA sequences (access #: NM_001044633.1 and NM_000384.3 in Genbank, respectively). Results showed 3 identical segments at 8, 7, and 5-bp length matched to the sequence of chicken and human apoB mRNA
